# A radiomics-based model for predicting lymph nodes metastasis of pancreatic ductal adenocarcinoma: a multicenter study

**DOI:** 10.1186/s13244-025-02025-2

**Published:** 2025-06-27

**Authors:** Liwen Zhu, Ben Zhao, Tianyi Xia, Di Chang, Cong Xia, Mengqiu Liu, Ridong Li, Buyue Cao, Yue Qiu, Yaoyao Yu, Shuwei Zhou, Huayu Chen, Wu Cai, Zhimin Ding, Chunqiang Lu, Tianyu Tang, Yang Song, Yuancheng Wang, Jing Ye, Ying Liu, Shenghong Ju

**Affiliations:** 1 Nurturing Center of Jiangsu Province for State Laboratory of AI Imaging & Interventional Radiology, Department of Radiology, Zhongda Hospital, Medical School of Southeast University, Nanjing, China; 2 Department of Radiology, The First Affiliated Hospital of University of Science and Technology of China, Hefei, China; 3 Department of Radiology, Northern Jiangsu People’s Hospital, Yangzhou, China; 4 Department of Radiology, The Second Affiliated Hospital of Soochow University, Suzhou, China; 5 Department of Radiology, Yijishan Hospital of Wannan Medical College, Wuhu, China; 6 MR Scientific Marketing, Siemens Healthineers, Shanghai, China

**Keywords:** Pancreatic ductal adenocarcinoma, Lymph nodes metastasis, Radiomics, Computed tomography, Prognosis

## Abstract

**Purpose:**

To develop a radiomics model to predict lymph nodes metastasis (LNM) in patients with pancreatic ductal adenocarcinoma (PDAC) and assess its value for clinical management.

**Methods:**

Patients with pathologically confirmed PDAC from four centers were retrospectively enrolled and split into four cohorts: training (*n* = 192), validation (*n* = 82), testing (*n* = 100), and clinical utilization (*n* = 163). A radiomics model was constructed based on contrast-enhanced CT (CECT) to predict LNM, and its performance was evaluated using the areas under the curve (AUC). Kaplan–Meier analysis was used to assess the prognostic and therapeutic decision-assisting value of the radiomics model.

**Results:**

A total of 437 patients (mean age: 63.1 years ± 9.2 standard deviation; 253 men) were included. The radiomics model outperformed other models with AUCs of 0.84, 0.82, and 0.78 in the training, validation, and testing cohorts (all *p* < 0.05), respectively. LNM predicted by the radiomics model was significantly associated with overall survival (*p* < 0.001). Kaplan–Meier analysis revealed that patients with a higher risk of LNM also had worse outcomes (all *p* < 0.05). Additionally, among the high-risk subgroup identified by the radiomics model in the clinical utilization cohort, patients who underwent dissection of ≥ 15 lymph nodes exhibited better overall survival compared to those with fewer lymph nodes dissected (*p* = 0.002).

**Conclusion:**

The radiomics model we constructed demonstrated impressive performance in predicting LNM and prognosis, suggesting its potential for optimizing the clinical management of PDAC.

**Critical relevance statement:**

This radiomics model can predict the risk of lymph nodes metastasis and prognosis of patients in pancreatic ductal adenocarcinoma and has potential value in selecting patients who can benefit from different extents of lymph nodes dissection.

**Key Points:**

- Thorough lymph node dissection is important for achieving the best prognosis in pancreatic ductal adenocarcinoma (PDAC).
- The radiomics model can accurately predict lymph node status and stratify patients’ prognosis.
- This radiomics model enhances the clinical management of PDAC.

**Graphical Abstract:**

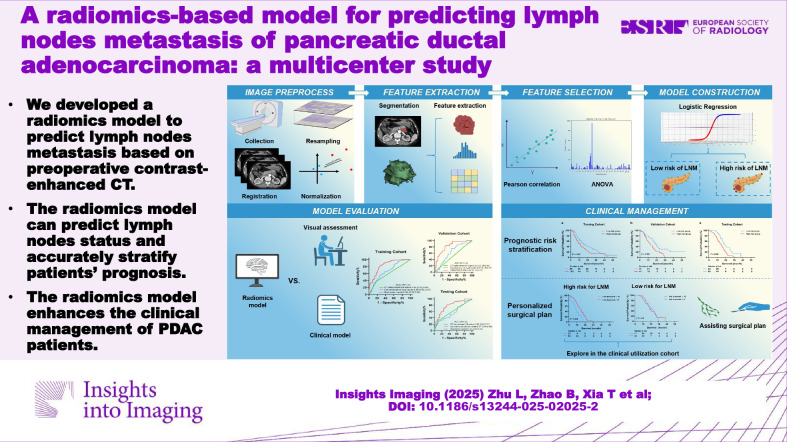

## Introduction

Pancreatic ductal adenocarcinoma (PDAC) remains one of the most malignant tumors with a 5-year survival rate of approximately 13% [1]. Rapid progression and early-stage lymph nodes metastasis (LNM) is a hallmark of PDAC, which has been identified as an independent predictor of a poor prognosis [2–4]. Performing a thorough removal of metastatic lymph nodes (LNs) is critical for surgical management and can significantly impact patient outcomes. The NCCN guidelines recommend that at least 15 LNs should be dissected. However, despite undergoing more extensive LNs dissection, survival rates remain low, and a subset of patients unfortunately experience early postoperative mortality due to an increased incidence of complications [5, 6]. This suggests that the number of LNs removed may not be the sole determinant of outcome; rather, the extent of dissection should strike a balance between the risk of LNM and the potential for increased postoperative complications. Additionally, recent studies indicate that neoadjuvant therapy has the potential to optimize patients’ outcomes, particularly for those at high risk of LNM [7, 8]. Consequently, accurate preoperative assessment of LNs status is crucial for determining optimal surgical strategy and developing personalized treatment plans.

Determining the risk of LNM prior to surgery in PDAC remains a significant challenge. Traditionally, the assessment of LN involvement mainly relied on the subjective evaluation by radiologists, which lacks sensitivity and accuracy [9, 10]. Additionally, several studies have used serological or genetic biomarkers as predictive indicators for LNM, but their application has been limited by low efficacy and high costs [11, 12]. Therefore, there is an urgent need for a novel technique that can accurately predict LN status before surgery.

Radiomics extracts complex and multi-dimensional features from medical images through automated or semi-automated analysis [13, 14]. It has been widely employed in oncological research and considered a key tool in precision medicine [15, 16]. Radiomics has been applied in various solid tumor types to predict LNM, including PDAC. However, most of these studies had small sample sizes and were conducted in a single center, limiting their reproducibility [17–20]. Moreover, to our knowledge, no study has explored the utility of radiomics models in identifying patients who would benefit from more extensive LN dissections [21].

Therefore, we aim to develop a radiomics model based on contrast-enhanced computed tomography (CECT) to predict LNM preoperatively and explore its predictive value in patient prognosis. Furthermore, we explore the model’s ability to identify patients who would benefit from dissecting more LNs in a retrospective cohort.

## Methods

### Patients

**Fig. 1:**
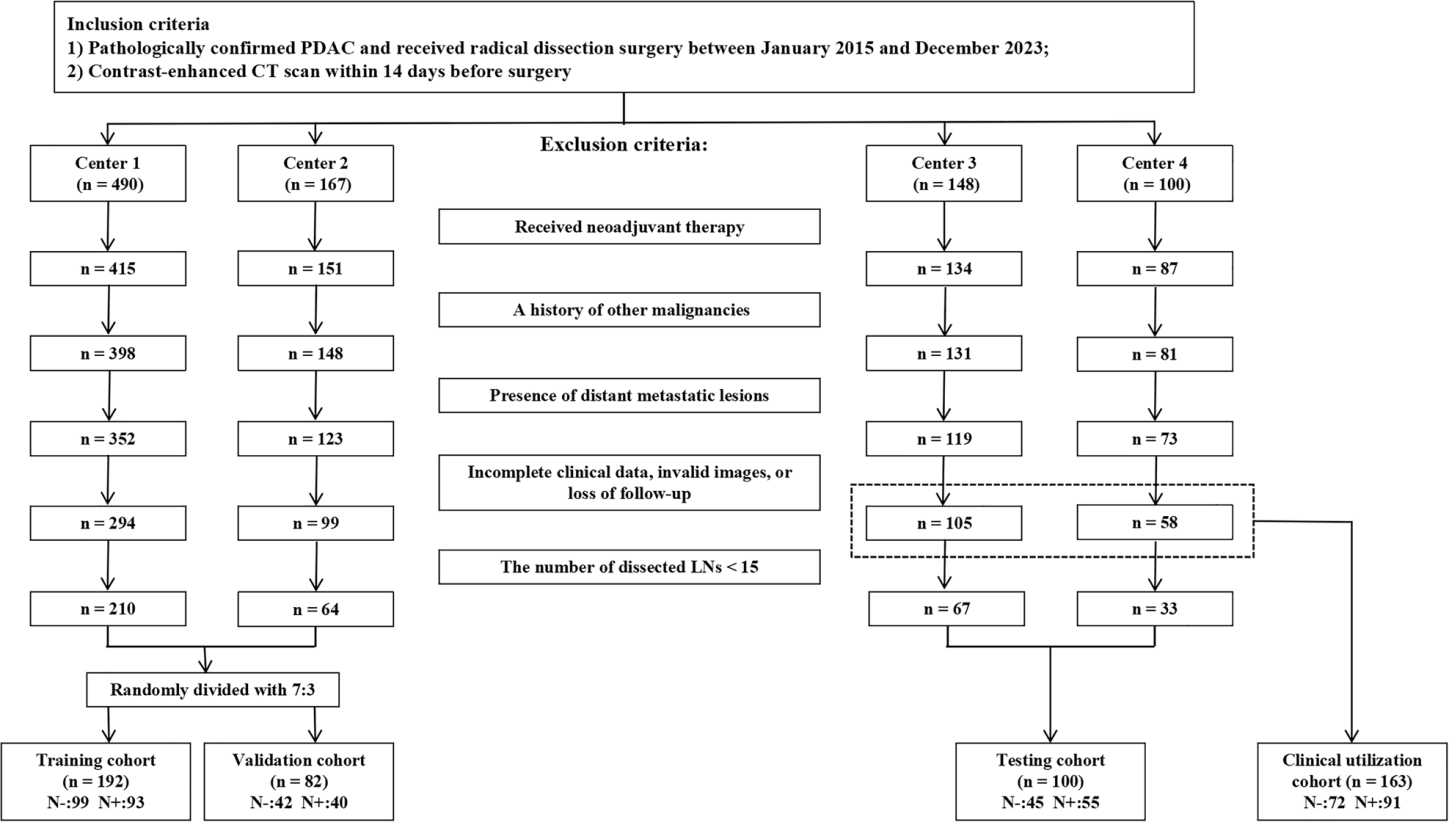
Overview of the patient selection process from four medical centers and data distribution

This multicenter retrospective study was approved by the Research Ethics Committee of the Institutional Review Boards, and the requirement for written informed consent was waived. Consecutive patients diagnosed with PDAC by postoperative pathological examination between January 2015 and December 2023 were enrolled from four centers: the First Affiliated Hospital of the University of Science and Technology of China (Center 1), the Yijishan Hospital of Wannan Medical College (Center 2), the Northern Jiangsu People’s Hospital (Center 3), and the Second Affiliated Hospital of Soochow University (Center 4). All patients included in the study underwent primary radical resection according to National Comprehensive Cancer Network (NCCN) guidelines. Based on the fundamental principles of performing pancreaticoduodenectomy for tumors in the head and neck of the pancreas and distal pancreatectomy for tumors in the body and tail, individualized resections were conducted according to the actual extent of tumor infiltration. The inclusion criteria were: (1) pathologically confirmed PDAC and received radical dissection surgery; (2) contrast-enhanced CT scan within 14 days before surgery. Patients were excluded if they had: (1) received neoadjuvant therapy; (2) a history of other malignancies; (3) presence of distant metastatic lesions; (4) incomplete clinical data, invalid images, or were lost to follow-up; (5) the number of dissected LNs < 15. Additionally, patients from Centers 3 and 4 who had < 15 LNs dissected were included in a clinical utilization cohort alongside those with ≥ 15 LNs dissected. A random 7:3 split was applied to patients from Centers 1 and 2 to form training and validation cohorts, respectively. Meanwhile, patients from Centers 3 and 4 who had ≥ 15 LNs dissected constituted an independent testing cohort (Fig. 1). Follow-up was conducted every 3 months for the first 2 years post-surgery and every 6 months thereafter. The last follow-up of patients was conducted on June 30, 2024. Overall survival (OS) was defined as the time from surgery to death or the last follow-up.

### Data collection

Baseline clinical data, including age, gender, total bilirubin, direct bilirubin, albumin, and serum carbohydrate antigen 19-9 (CA19-9), were extracted from electronic medical records. Pathological data were obtained from reports, including tumor differentiation, number of LNs dissected, number of metastatic LNs, perineural invasion status, lymphovascular invasion status, and resection margin status. The ground truth of LN status was determined based on the postoperative pathological report. Relevant information regarding CECT images is shown in the Supplementary Material.

### Image assessment

Blinded to the radiological and pathological reports, two radiologists (L.Z. and B.Z., with 3 and 5 years’ clinical experience, respectively) assessed the CECT scans to document the location and size of the tumor. CT-determined LN status was defined as classification evaluated by radiologists based on five criteria: short-axis diameter > 10 mm, nonuniform enhancement, inhomogeneous density, internal necrosis, LN fusion, or blurred borders. Any discrepancies were resolved by a senior radiologist (C.X., with 7 years of clinical experience) to achieve a final consensus reading.

### Model development and validation

**Fig. 2:**
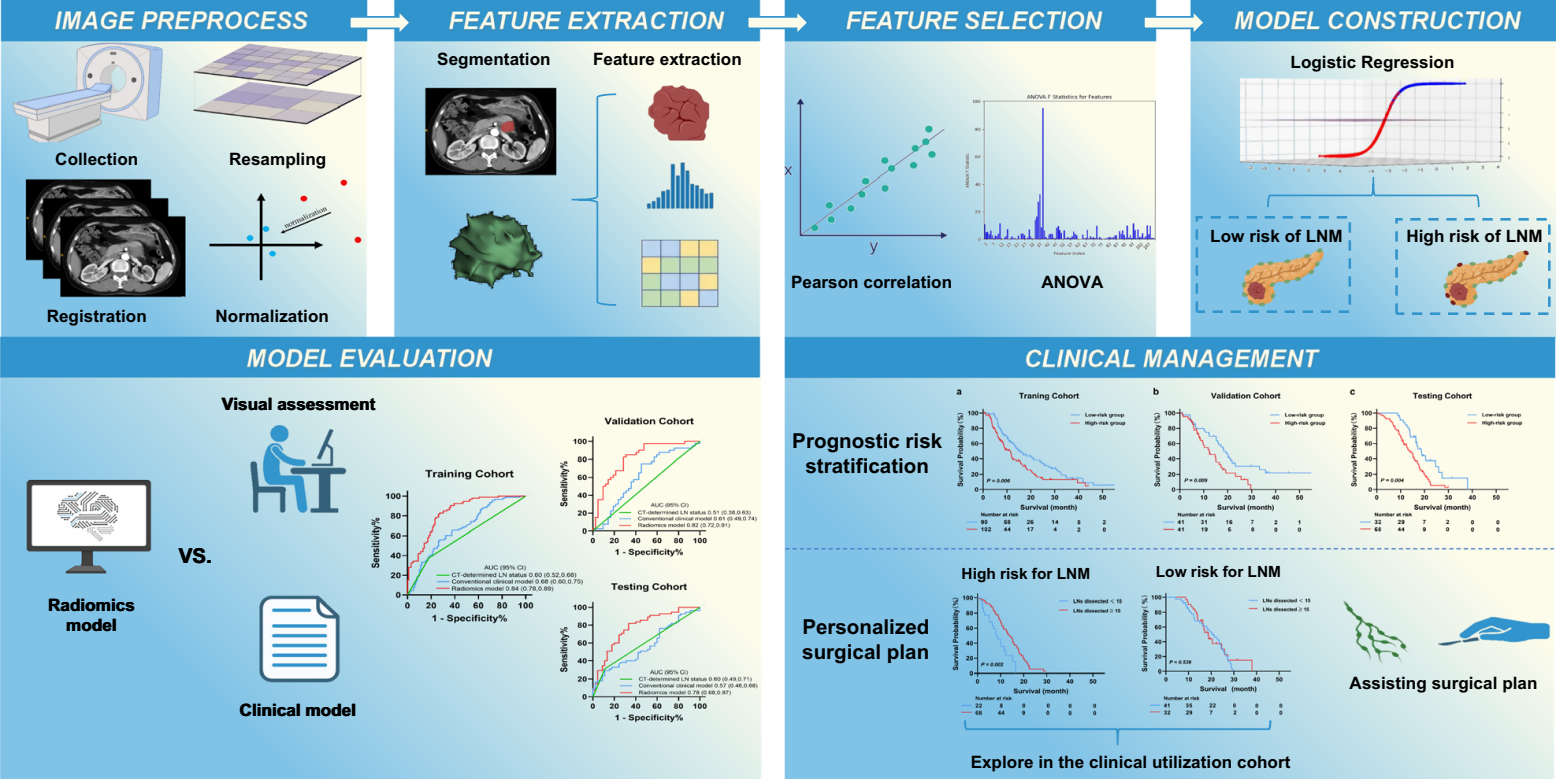
Radiomics workflow and study flowchart. LNM, lymph node metastasis

The primary tumor of PDAC was automatically segmented using a technique based on the nnU-Net architecture [22, 23]. One of the two abdominal radiologists (C.X. and B.Z.) reevaluated and manually modified the segmentation results. Subsequently, we extracted radiomics features from the arterial phase of CECT scans according to the Imaging Biomarker Standardization Initiative standards, and finally, 106 features were extracted [24]. Analysis of variance was utilized to select features based on the F-value, and the Pearson correlation coefficient was applied to eliminate redundant features at a threshold value of 0.99. The radiomics model was constructed by logistic regression. All the processes above were conducted by Feature Explorer (version 0.4.0), an open-source software based on PyRadiomics [25]. Predictive performance of the radiomics model was evaluated in the validation and testing cohorts. A conventional clinical model was developed using logistic regression based on several preoperative variables, including tumor size, location, CA19-9, and CT-determined LN status. Diagnostic accuracy among the radiomics model, conventional clinical model, and independent CT-determined LN status was compared. Furthermore, the robustness of the radiomics model was also examined in subgroups defined by tumor size ≤ 3 cm and > 3 cm. The radiomics workflow and study flowchart are shown in Fig. 2. To address the adherence to good practice in radiomics research, we evaluated our study using the Radiomics Quality Score (RQS) criteria, and a detailed scoring table based on the RQS criteria is provided in Supplementary Table 6 [15].

### Prognostic value of the radiomics model

Given the significant impact of LNM on the prognosis of PDAC, the radiomics model’s prognostic value was further investigated. First, the optimal cutoff value for the Rad-score, a measure representing the probability of LNM calculated by the radiomics model, was determined through Youden’s index in the training cohort. Subsequently, patients in training, validation, and testing cohorts were categorized into high-risk or low-risk for LNM according to the same cutoff value. Univariable and multivariable Cox regression analyses were used to evaluate the prognostic predictive performance of the model. Kaplan–Meier analysis combined with log-rank test was employed to compare OS between low-risk and high-risk subgroups in the training, validation, and testing cohorts.

### Clinical utility of the radiomics model

To investigate the efficacy of this radiomics model in identifying patients who can benefit from more extensive LN dissection. Patients from Centers 3 and 4, regardless of the number of dissected LNs, were enrolled to compose a clinical utilization cohort. First, patients were divided into high-risk and low-risk groups based on the previously determined optimal cutoff value for the Rad-score. Subsequently, OS was compared between patients with ≥ 15 LNs dissected and those with < 15 LNs within each risk subgroup. The threshold of 15 LNs was selected based on existing clinical guidelines and previous studies that suggest this number as a critical benchmark for adequate staging and prognosis in PDAC [26].

### Statistical analysis

Continuous data with a normal distribution are presented as mean ± standard deviation (SD); otherwise, the median and interquartile range (IQR) are used. Categorical variables are presented as numbers and percentages (*n*, %). Continuous variables were tested using the Wilcoxon test, while categorical variables were tested using the chi-square and Fisher’s exact test. Area under the curve (AUC), sensitivity, specificity, and accuracy were used to evaluate the predictive performance of the radiomics model, conventional clinical model, and CT-determined LN status. AUC values were compared among three models using the Delong test. A calibration plot was used to align expected and observed probabilities of LNM. Decision curve analysis (DCA) was used to measure the net benefit derived from the radiomics model across various threshold probabilities. The association between the results predicted by the radiomics model and OS was examined using univariable and multivariable Cox regression analyses. Kaplan–Meier analysis and log-rank test were used to compare OS differences among patients categorized by different risks of LNM. *p* < 0.05 was regarded as statistically significant. All statistical tests were two-sided. R (version 4.2.2) was used to analyze the data, while GraphPad Prism (version 8.0.2) was used to visualize the results.

## Results

### Demographic and pathological characteristics

A total of 437 consecutive patients (mean age: 63.1 years ± 9.2 [SD]; 253 men) with pathologically confirmed PDAC were enrolled in this study, of whom 374 (mean age: 63.0 years ± 6.9 [SD]; 218 men) had ≥ 15 LNs dissected. These patients were divided into three cohorts: training (*n* = 192), validation (*n* = 82), and testing (*n* = 100). There were 188 of 374 patients with LNM, with no significant difference observed in the proportion of LNM-positive patients across the three cohorts (*p* > 0.05). The detailed demographic and clinical-pathological characteristics of each cohort are shown in Table 1. Furthermore, Supplementary Tables 1–3 provide an in-depth comparison of clinical-pathological features between patients with and without LNM within each cohort.

**Table 1:** The demographic and pathological characteristics of patients in the training, validation, and testing cohorts

Variables	Training cohort (*n* = 192)	Validation cohort (*n* = 82)	Testing cohort (*n* = 100)
Age (years)^a^	62.6 ± 9.1	62.4 ± 9.2	64.0 ± 9.0
Gender			
Female	80 (41.7)	31 (37.8)	45 (45.0)
Male	112 (58.3)	51 (62.2)	55 (55.0)
Tumor location			
Head and neck	158 (82.3)	57 (69.5)	79 (79.0)
Body and tail	34 (17.7)	25 (30.5)	21 (21.0)
Tumor size (cm)^b^	3.5 (3.0, 4.0)	3.3 (2.8, 4.5)	3.1 (2.2, 3.5)
CA19-9 (U/mL)			
≤ 210	86 (44.8)	49 (59.8)	49 (49.0)
> 210	106 (55.2)	33 (40.2)	51 (51.0)
Total bilirubin (μmol/L)^b^	60.1 (14.5, 204.0)	18.8 (11.7, 152.8)	24.2 (12.3, 123.2)
Direct bilirubin (μmol/L)^b^	35.8 (5.0, 144.5)	7.4 (4.4, 111.4)	9.6 (4.1, 105.8)
Albumin (g/L)^b^	40.2 (37.0, 43.3)	40.3 (37.3, 44.2)	44.2 (40.4, 46.0)
Differentiation			
Well or moderate	90 (46.9)	38 (46.3)	82 (82.0)
Poor	102 (53.1)	44 (53.7)	18 (18.0)
T stage			
T1	20 (10.4)	12 (14.6)	22 (22.0)
T2	131 (68.2)	48 (58.5)	67 (67.0)
T3	41 (21.4)	22 (26.8)	11 (11.0)
TNM stage			
Stage I	96 (50)	37 (45.1)	45 (45.0)
Stage II	90 (46.9)	39 (47.6)	44 (44.0)
Stage III	6 (3.1)	6 (7.3)	11 (11.0)
Nerve infiltration			
Absent	44 (22.9)	24 (29.3)	19 (19.0)
Present	148 (77.1)	58 (70.7)	81 (81.0)
Lymphovascular invasion			
Absent	137 (71.4)	63 (76.8)	77 (77.0)
Present	55 (28.6)	19 (23.2)	23 (23.0)
Resection region			
R0	184 (95.8)	76 (92.7)	94 (94.0)
R1	8 (4.2)	6 (7.3)	6 (6.0)
LNM			
Absent	99 (51.6)	42 (51.2)	45 (45.0)
Present	93 (48.4)	40 (48.8)	55 (55.0)

Except where indicated, data are numbers of patients, with percentages in parentheses

*LNM* lymph node metastasis, *CA19-9* carbohydrate antigen 19-9 (U/mL), *R0* negative surgical margin, *R1* positive surgical margin

^a^ Mean ± standard deviation

^b^ Median (interquartile range)

### The prediction performance of LNM

**Fig. 3:**
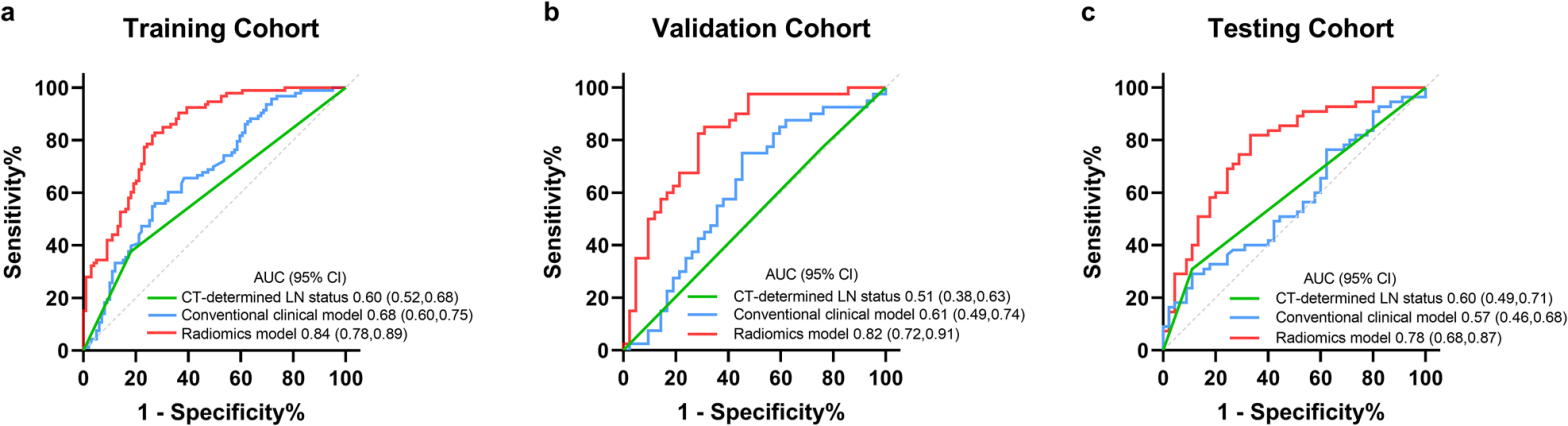
AUC values of the radiomics model, conventional clinical model, and CT-determined LN status for predicting lymph node metastasis in the training cohort (**a**), validation cohort (**b**), and testing cohort (**c**). AUC, the area under the curves; 95% CI, 95% confidence interval; LN, lymph node. AUCs were reported with 95% CI in parentheses

The radiomics model was finally constructed with 10 radiomics features. AUCs for the training, validation, and testing cohorts were 0.84 (95% CI: 0.78, 0.89), 0.82 (95% CI: 0.72, 0.91), and 0.78 (95% CI: 0.68, 0.87), respectively, demonstrating consistently strong predictive accuracy. Calibration curves revealed a close alignment between predicted LNs status and actual measurements (Supplementary Fig. 1). In all three cohorts, the AUC values of the radiomics model were significantly higher than those of the CT-determined LN status and the conventional clinical model (all *p* < 0.05) (Fig. 3). The sensitivity, specificity, and accuracy of the radiomics model for the independent testing cohort were 85.5%, 53.3%, and 71.0% respectively, outperforming both the CT-determined LN status and the conventional clinical model in terms of sensitivity and accuracy (Table 2). The DCA curves demonstrated the great clinical utility of the radiomics model in each cohort (Supplementary Fig. 2). Additionally, subgroup analysis based on tumor size revealed that the model maintained strong performance in both subgroups, with an AUC of 0.74 (95% CI: 0.65, 0.82) for tumors ≤ 3 cm and an AUC of 0.85 (95% CI: 0.80, 0.89) for tumors > 3 cm (Supplementary Fig. 3).

**Table 2:** Performance of the radiomics model, the conventional clinical model, and CT-determined LN status

Model	Cohort	AUC^a^	Sensitivity	Specificity	Accuracy
Radiomics	Training	0.84 (0.78, 0.89)	81.7 (76/93)	73.7 (73/99)	77.6 (149/192)
	Validation	0.82 (0.72, 0.91)	72.5 (29/40)	71.4 (30/42)	72.0 (59/82)
	Testing	0.78 (0.68, 0.87)	85.5 (47/55)	53.3 (24/45)	71.0 (71/100)
Conventional clinical	Training	0.68 (0.60, 0.75)	55.9 (52/93)	72.7 (72/99)	64.6 (124/192)
	Validation	0.61 (0.49, 0.74)	37.5 (15/40)	71.4 (30/42)	54.9 (45/82)
	Testing	0.57 (0.46, 0.68)	49.1 (27/55)	57.8 (26/45)	53.0 (53/100)
CT-determined LN status	Training	0.60 (0.52, 0.68)	37.6 (35/93)	81.8 (81/99)	60.4 (116/192)
	Validation	0.51 (0.38, 0.63)	22.5 (9/40)	76.2 (32/42)	50.0 (41/82)
	Testing	0.60 (0.49, 0.71)	30.9 (17/55)	88.9 (40/45)	57.0 (57/100)

Except where indicated, data are percentages (%), with numbers of patients in parentheses

*AUC* area under the receiver operating characteristic curve

^a^ Data are AUCs, with 95% confidence intervals (95% CIs) in parentheses

### Prognostic value of the radiomics model

The optimal cutoff value of Rad-score was determined to be 0.45 using the maximum Youden index from the training cohort. Patients from three cohorts with a Rad-score ≥ 0.45 were categorized into a high-risk subgroup, while those with a Rad-score < 0.45 were assigned to the low-risk subgroup. Univariable Cox regression analysis revealed that the high-risk subgroup was significantly associated with poorer outcomes (hazard ratio (HR): 1.76; 95% CI: 1.38, 2.25; *p* < 0.001). Furthermore, after adjusting for clinical variables, multivariable Cox regression analysis revealed that LNM, as predicted by the radiomics model, remained an independent predictor of OS (HR: 1.60; 95% CI: 1.24, 2.06; *p* < 0.001) (Table 3).

**Table 3:** Univariable and multivariable Cox regression analysis of overall survival

Variables	Univariable analysis		Multivariable analysis	
	HR (95% CI)	*p*-value	HR (95% CI)	*p*-value
Risk-stratification				
Low-risk	Reference		Reference	
High-risk	1.76 (1.38, 2.25)	< 0.001^a^	1.60 (1.24, 2.06)	< 0.001^b^
Age (years)	1.02 (1.00, 1.03)	0.013^a^	1.02 (1.00, 1.03)	0.032^b^
Gender				
Female	Reference			
Male	0.97 (0.76, 1.23)	0.778		
Tumor location				
Head and neck	Reference			
Body and tail	0.83 (0.62, 1.11)	0.209		
Tumor size (cm)	1.03 (0.94, 1.12)	0.553		
CA19-9 (U/mL)				
≤ 210	Reference		Reference	
> 210	1.24 (0.98, 1.57)	0.078^a^	1.14 (0.89, 1.46)	0.308
Total bilirubin (μmol/L)	1.00 (1.00, 1.00)	0.539		
Direct bilirubin (μmol/L)	1.00 (1.00, 1.00)	0.228		
Differentiation				
Well or moderate	Reference			
Poor	1.18 (0.93, 1.52)	0.189		
T stage				
T1	Reference		Reference	
T2	1.74 (1.20, 2.52)	0.003^a^	1.70 (1.16, 2.49)	0.006^b^
T3	1.39 (0.90, 2.14)	0.142	1.12 (0.68, 1.83)	0.665
TNM stage				
Stage I	Reference		Reference	
Stage II	1.74 (1.20, 2.52)	0.001^a^	1.58 (1.17, 2.14)	0.003^b^
Stage III	2.28 (1.41, 3.68)	0.001^a^	1.84 (1.12, 3.01)	0.016^b^
Nerve infiltration				
Absent	Reference			
Present	1.26 (0.95, 1.67)	0.107		
Lymphovascular invasion				
Absent	Reference		Reference	
Present	1.50 (1.14, 1.97)	0.004^a^	1.52 (1.13, 2.03)	0.005^b^
Resection region				
R0	Reference			
R1	1.49 (0.90, 2.48)	0.120		
CT-determined LN status				
Absent	Reference		Reference	
Present	1.45 (1.11, 1.90)	0.007^a^	1.30 (0.99, 1.72)	0.062

Risk-stratification was determined by Rad-score calculated by the radiomics model according to the optimal cutoff value

Variables for which ^a^ *p* < 0.1 in the univariable analysis were included in the multivariable analysis, while ^b^ *p* < 0.05 in the multivariable analysis was regarded as statistically significant

*CA19-9* carbohydrate antigen 19-9 (U/mL), *R0* negative surgical margin, *R1* positive surgical margin, *HR* hazard ratio, *CI* confidence interval

**Fig. 4:**
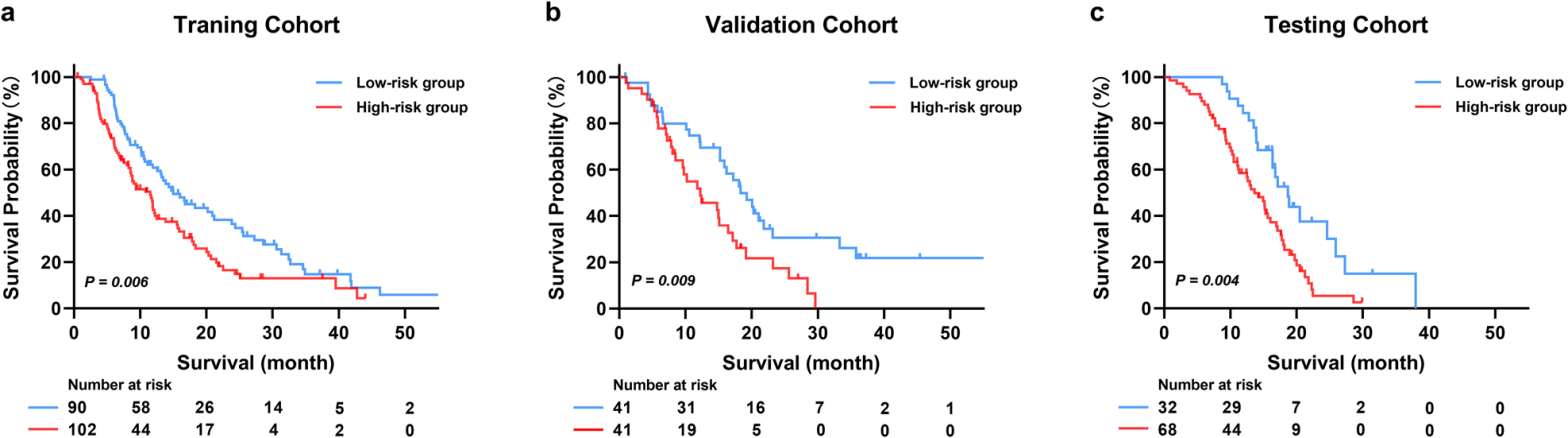
Kaplan–Meier analysis of overall survival between patients with different risks of LNM in the training (**a**), validation (**b**), and testing (**c**) cohorts. *p*-values were calculated using the log-rank test

Kaplan–Meier analysis suggested patients predicted to have a high risk of LNM had a shorter OS compared to those with low risk (median OS: 11.5 vs. 15.0 months, *p* = 0.006) in the training cohort, validation cohort (median OS: 12.2 vs. 18.3 months, *p* = 0.009), and testing cohort (median OS: 13.6 vs. 18.7 months, *p* = 0.004) (Fig. 4). Subgroup analysis according to different tumor sizes also showed similar results that patients with a high risk of LNM had worse prognosis (all *p* < 0.05) (Supplementary Fig. 4).

### Clinical utility of the radiomics model

**Fig. 5:**
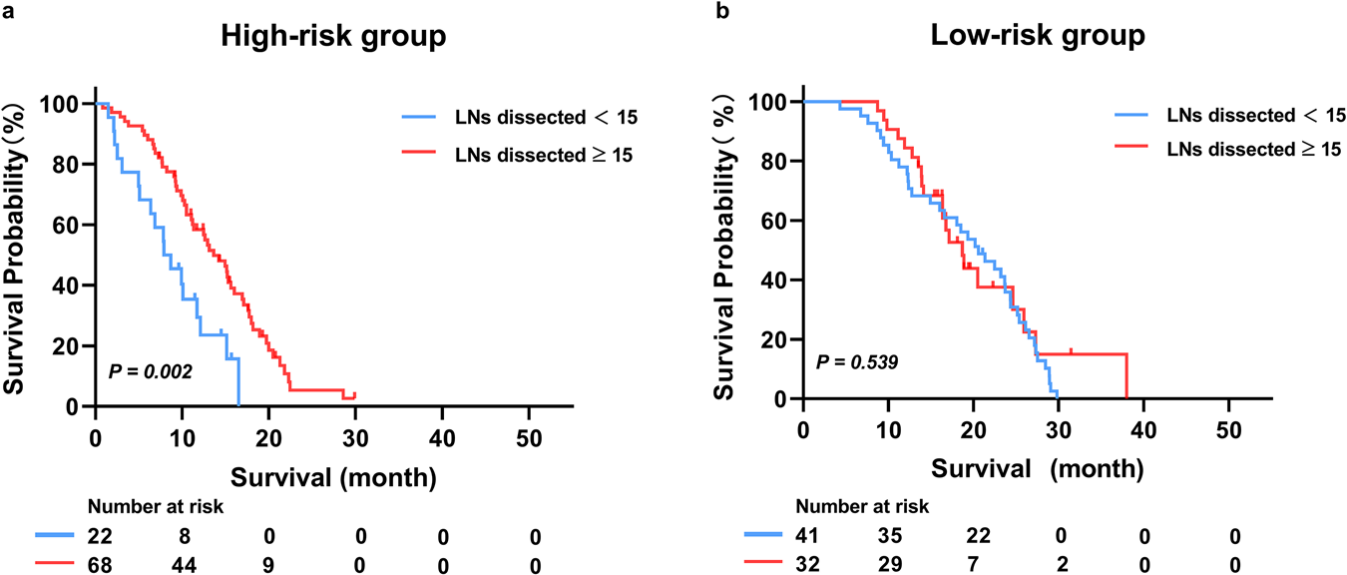
Kaplan–Meier analysis of overall survival between patients with different numbers of dissected LNs in the high-risk group (**a**) and low-risk group (**b**) predicted by the radiomics model. *p*-values were calculated using the log-rank test. LNs, lymph nodes

To further evaluate the clinical utility of the radiomics model, a retrospective cohort of 163 patients (mean age: 64.0 ± 9.0 [SD]; 90 men) from Centers 3 and 4 was analyzed. The detailed demographic and pathological characteristics are shown in Supplementary Table 4. Patients were stratified into high-risk and low-risk subgroups based on the same cutoff value. Survival analysis revealed that patients with ≥ 15 LNs dissected had better OS than those with < 15 LNs dissected (median OS, 13.6 vs. 8.3 months, *p* = 0.002). Conversely, no difference was observed in the low-risk subgroup between patients with ≥ 15 LNs dissected and those with < 15 LNs dissected (median OS: 18.7 vs. 20.6 months, *p* = 0.539) (Fig. 5).

## Discussion

In this retrospective multicenter study, we developed a radiomics model based on preoperative CECT to predict LNM in PDAC. Our findings indicated that the radiomics model outperformed the conventional clinical model, which includes a number of preoperative clinical features and the CT-determined LN status assessed visually by radiologists. Furthermore, it also demonstrated robust predictive efficacy in subgroup analyses across different tumor sizes, and a significant association was identified between the prognostic outcome divided by the radiomics model and OS. Our study revealed a potential association between the number of LNs dissected and OS among patients classified as high-risk by the radiomics model. This finding may help surgeons select patients who will benefit from dissecting more LNs.

Accurate preoperative identification of LNs status is essential to tumor staging and determining individualized treatment strategies [27, 28]. Radiologists’ subjective assessment based on medical images has been the primary method for evaluating LN status preoperatively [29]. However, studies have shown that radiologists’ subjective assessment can only screen out a limited proportion of patients with obvious diseases while lacking reproducibility and consistency. Although positron emission tomography-computed tomography offers more precise identification for metastatic LNs, its application is restricted due to high costs and limited availability [30]. Our radiomics model, based on arterial phase images, achieved a sensitivity of 85.5% in detecting LNM among PDAC patients in the testing cohort, indicating its potential as a valuable tool for preoperative identification of patients with actual metastatic involvement. The high sensitivity of our model is crucial, as it enables the identification of patients who may have a significant survival benefit from personalized treatments. However, the specificity of 53.3% reflects a limitation that must be considered, which implies a higher likelihood of false positives. This potentially leads to an unnecessarily extensive LNs dissection. The low specificity could be attributed to the lack of features from LNs. Future research efforts will focus on enhancing the specificity of our model, achieving automated segmentation of peripancreatic LNs, and integrating LNs-specific radiomic features. Overall, the radiomics model we have developed is a useful tool for preoperatively identifying patients with metastatic LNs, demonstrating the value of radiomics for reflecting the invasive characteristics of PDAC [31, 32].

Despite recent improvements in the treatment of PDAC, the prognosis for PDAC remains poor [1]. Accurate prognostication is essential for making personalized treatment strategies. Although the pathological TNM staging system is a valuable tool for estimating survival outcomes, its application is delayed until postoperative pathological analysis [33, 34]. In contrast, the radiomics model proposed in this study offers a preoperative prognostic assessment, enabling timely identification of patients at high risk of LNM and facilitating individualized treatment planning, especially in selecting individuals who are likely to derive substantial benefit from neoadjuvant therapies [35]. At the same time, our model can emphasize the necessity for regular postoperative surveillance and the timely initiation of consolidative treatments through prognosis prediction [36, 37].

Tumor-draining LNs play an important role in modulating the host’s anti-tumor response and boosting the effectiveness of immunotherapies [38–40]. Consequently, the extent of LNs dissection during surgery is a topic of significant interest, particularly in the context of individualized treatment planning. We explored whether there is a relationship between the risk of LNM predicted by our model and the number of LNs dissected. Surprisingly, we found that the survival discrepancies were linked to the number of LNs dissected in the high-risk subgroup predicted by the radiomics model. Specifically, patients with ≥ 15 LNs dissected in the high-risk subgroup had longer OS than those with < 15 LNs dissected; in contrast, the low-risk subgroup showed no such association. This finding indicates that patients identified as high risk for LNM by our model might achieve improved survival outcomes with more extensive LN dissection. In future, with the advancement in AI and medicine, our proposed model could be integrated into clinical practice after prospective validation. It could serve as a feasible tool in the routine radiological workflow. This integration allows for fast, accurate assessment of the risk of LNM, thereby enhancing clinical decision-making.

The multicenter retrospective study achieved an RQS of 18, indicating there are still some limitations in our study. First, the retrospective design introduces selection bias, and although the study is multicentric, the sample size remains limited. Second, while the radiomics model is designed to estimate the risk of LNM on an individual basis, it does not provide insights into the exact numbers or concrete sites of the affected LNs. In the future, we hope to collaborate more closely with surgeons and pathologists to achieve a correspondence between surgically resected LNs and imaging data, which would enable our model to develop the capability to localize metastatic LNs. Third, the relatively lower specificity of the model increases the likelihood of false positives, potentially leading to unnecessary treatments. Fourth, the lack of biological interpretability of radiomic features hinders the model’s clinical applicability. Future research should aim to bridge this gap by integrating radiomic findings with biological and molecular data to enhance the model’s reliability. Finally, prospective validation in a larger, multicentric cohort is necessary to fully establish the clinical utility of the radiomics model.

To sum up, this study demonstrated that the radiomics model based on preoperative CECT is an effective method for predicting LNM in patients with PDAC. The radiomics model outperformed both the conventional clinical model and radiologists’ subjective assessment. Furthermore, this approach holds potential for prognosis prediction and personalized surgical management.

## Supplementary information

ELECTRONIC SUPPLEMENTARY MATERIAL

## Data Availability

The datasets generated and/or analyzed during the current study contain sensitive personal information and are therefore not publicly available to protect the privacy and confidentiality of the individuals involved. However, de-identified datasets may be available from the corresponding author upon reasonable request and with permission from the relevant ethics committee, ensuring compliance with all legal and ethical requirements.

## Abbreviations

AUC: Area under the curve
CT: Computed tomography
CI: Confidence interval
CECT: Contrast-enhanced computed tomography
DCA: Decision curve analysis
HR: Hazard ratio
LNM: Lymph nodes metastasis
LNs: Lymph nodes
OS: Overall survival
PDAC: Pancreatic ductal adenocarcinoma
SD: Standard deviation
