# Supplementary material for: A radiomics-based model for predicting lymph nodes metastasis of pancreatic ductal adenocarcinoma: a multicenter study
Source: Insights Imaging. 2025 Jun 27;16:141. doi: 10.1186/s13244-025-02025-2 (PMC12204970; doi:10.1186/s13244-025-02025-2)

A Radiomics-based Model for Predicting Lymph Nodes Metastasis of Pancreatic Ductal Adenocarcinoma: A Multi-center Study

ELECTRONIC SUPPLEMENTARY MATERIAL

Supplemental text

CT imaging acquisition and protocol

All patients enrolled into this study had standardized abdominal contrast-enhanced CT from respective hospital. CT scans were performed for all patients using one of the following systems: Brilliance 64, Optima CT 660, Discovery CT 750 HD, Revolution CT, Optima CT 680 Series, SOMATOM Definition, Aquilion ONE, uCT 510, uCT 780, LightSpeed VCT, iCT 256, and IQon-Spectral CT. The scanning parameters were as follows: tube voltage of 120 kV; tube current of 50 - 400 mAs; the field of view, 350 × 350 - 500 × 500 mm; slice thickness, 5 mm. A 1.3 - 1.5 mL/kg body weight bolus of contrast material was injected intravenously at a flow rate of 3.0 - 4.0 mL/sec. Using a bolus-tracking technique, arterial phase scanning started 25 - 40 seconds after the descending aorta's enhancement hit 100 HU. Images of the portal venous phase were obtained 60 - 75 seconds after the contrast injected, and delay phase images were captured 90 - 120 seconds after the injection.

Methodology of extraction and selection of radiomic features

For the extraction of radiomic features, we adhered to the ISBI standards and extracted a total of 106 radiomic features from the tumor lesions identified in the arterial phase images, comprising 17 first-order statistics, 75 texture features, and 14 shape features.

We applied the normalization on the feature matrix. For each feature vector, we calculated the mean value and the standard deviation. Each feature vector was subtracted by the mean value and was divided by the standard deviation. After the normalization process, each vector has a zero center and unit standard deviation. Since the dimension of the feature space was high, we compared the similarity of each feature pair. If the PCC value of the feature pair was larger than 0.99, we removed one of them. After this process, the dimension of the feature space was reduced and each feature was independent of each other. Before building the model, we used analysis of variance (ANOVA) to select features. F-value was calculated to evaluate the relationship between features and the label. We sorted features according to the corresponding F-value and selected the sepcific number of features to build the model. We used logistic regression as the classifier. To determine the hyperparameter of the model, we applied cross-validation with 5-fold on the training data set. The hyperparameters were set according to the model’s performance on the validation dataset.

Supplemental Tables

Supplemental Table 1. The demographic and pathological characteristics of the training cohort.

Training Cohort (n = 192)			
Variables	LNM (n = 93)	Non-LNM (n = 99)	*P* Value
Age (years)[a]	63.2 ± 8.8	62.0 ± 9.4	0.269
Gender			0.942
Female	38 (40.9)	42 (42.4)	
Male	55 (59.1)	57 (57.6)	
Tumor location			0.003[c]
Head and neck	85 (91.4)	73 (73.7)	
Body and tail	8 (8.6)	26 (26.3)	
Tumor size (cm)[b]	3.5 (3.0, 4.0)	3.5 (3.0, 4.5)	0.105
CA19-9 (U/ml)			0.227
≤ 210	37 (39.8)	49 (49.5)	
> 210	56 (60.2)	50 (50.5)	
Total bilirubin (μmol/l)[b]	82.5 (16.8, 209.3)	43.9 (12.9, 186.1)	0.125

Direct bilirubin (μmol/l)[b]	47.1 (7.4, 149.6)	25.8 (4.6, 132.2)	0.443
Albumin (g/l)[b]	40.2 (35.1, 42.6)	40.2 (37.6, 43.8)	0.129
Differentiation			0.400
Well or moderate	47 (50.5)	43 (43.4)	
Poor	46 (49.5)	56 (56.6)	
T stage			0.040[c]
T1	9 (9.7)	11 (11.1)	
T2	71 (76.3)	60 (60.6)	
T3	13 (14.0)	28 (28.3)	
TNM stage			< 0.001[c]
Stage Ⅰ	31 (33.3)	65 (65.7)	
Stage Ⅱ	56 (60.2)	34 (34.3)	
Stage Ⅲ	6 (6.5)	0 (0.0)	
Nerve infiltration			0.533
Absent	19 (20.4)	25 (25.3)	
Present	74 (79.6)	74 (74.7)	
Lymphovascular invasion			0.553
Absent	64 (68.8)	73 (73.7)	
Present	29 (31.2)	26 (26.3)	
Resection region			0.652
R0	88 (94.6)	96 (97.0)	
R1	5 (5.4)	3 (3.0)	

CT-determined LN status			0.004[c]
Absent	58 (62.4)	81 (81.8)	
Present	35 (37.6)	18 (18.2)	
Rad-score[b]	0.62 (0.50, 0.86)	0.29 (0.16, 0.49)	< 0.001[c]

Note. Except where indicated, data are numbers of patients, with percentages in parentheses. CA19-9, carbohydrate antigen 19-9 (U/ml); R0, negative surgical margin; R1, positive surgical margin; LNM, lymph node metastasis.

[a] mean ± standard deviation, [b] median (interquartile range), [c] $P < 0.05$ is regarded statistically significant.

Supplemental Table 2. The demographic and pathological characteristics of the validation cohort.

	Validation Cohort (n = 82)		
Variables	LNM (n = 40)	Non-LNM (n = 42)	*P* Value
Age (years)[a]	63.6 ± 8.8	61.3 ± 9.6	0.275
Gender			0.460
Female	13 (32.5)	18 (42.9)	
Male	27 (67.5)	24 (57.1)	
Tumor location			0.076
Head and neck	32 (80.0)	25 (59.5)	
Body and tail	8 (20.0)	17 (40.5)	
Tumor size (cm)[b]	3.1 (2.5, 4.0)	3.5 (3.0, 4.5)	0.129
CA 19-9 (U/ml)			> 0.999
≤ 210	24 (60.0)	25 (59.5)	
> 210	16 (40.0)	17 (40.5)	
Total bilirubin (μmol/l)[b]	39.9 (11.9, 163.4)	16.1 (11.4, 99.6)	0.217
Direct bilirubin (μmol/l)[b]	25.0 (5.0, 123.3)	6.1 (4.3, 68.3)	0.180
Albumin (g/l)[b]	40.3 (36.5, 43.7)	40.3 (38.3, 45.2)	0.581
Differentiation			0.646

Well or moderate	17 (42.5)	21 (50.0)	
Poor	23 (57.5)	21 (50.0)	
T stage			0.683
T1	6 (15.0)	6 (14.3)	
T2	25 (62.5)	23 (54.8)	
T3	9 (22.5)	13 (30.9)	
TNM stage			0.005[c]
Stage Ⅰ	11 (27.5)	26 (61.9)	
Stage Ⅱ	24 (60.0)	15 (35.7)	
Stage Ⅲ	5 (12.5)	1 (2.4)	
Nerve infiltration			0.284
Absent	9 (22.5)	15 (35.7)	
Present	31 (77.5)	27 (64.3)	
Lymphovascular invasion			0.006[c]
Absent	25 (62.5)	38 (90.5)	
Present	15 (37.5)	4 (9.5)	
Resection region			0.627
R0	36 (90.0)	40 (95.2)	
R1	4 (10.0)	2 (4.8)	
CT - determined LN status			> 0.999
Absent	31 (77.5)	32 (76.2)	

Present	9 (22.5)	10 (23.8)	
Rad-score[b]	0.63 (0.44, 0.84)	0.19 (0.10, 0.48)	< 0.001[c]

Note. Except where indicated, data are numbers of patients, with percentages in parentheses. CA19-9, carbohydrate antigen 19-9 (U/ml); R0, negative surgical margin; R1, positive surgical margin; LNM: lymph nodes metastasis. [a] mean ± standard deviation, [b] median (interquartile range), [c] $P < 0.05$ is regarded statistically significant.

Supplemental Table 3. The demographic and pathological characteristics of the testing cohort.

	Testing Cohort (n = 100)		
Variables	LNM (n = 55)	Non-LNM (n = 45)	*P* Value
Age (years)[a]	63.8 ± 9.2	64.3 ± 8.8	0.788
Gender			0.189
Female	21 (38.2)	24 (53.3)	
Male	34 (61.8)	21 (46.7)	
Tumor location			0.980
Head and neck	44 (80.0)	35 (77.8)	
Body and tail	11 (20.0)	10 (22.2)	
Tumor size (cm)[b]	3.2 (2.3, 3.5)	3.1 (2.3, 3.5)	0.747
CA 19-9 (U/ml)			0.165
≤ 210	23 (41.8)	26 (57.8)	
> 210	32 (58.2)	19 (42.2)	
Total bilirubin (μmol/l)[b]	48 (12.6, 156.6)	16.7 (12.3, 104.6)	0.118
Direct bilirubin (μmol/l)[b]	34.4 (4.2, 127.3)	6.0 (4.0, 84.9)	0.157
Albumin (g/l)[b]	43.7 (40.1, 45.9)	44.7 (41.3, 46.4)	0.442

Differentiation			0.464
Well or moderate	47 (85.5)	35 (77.8)	
Poor	8 (14.5)	10 (22.2)	
T stage			0.758
T1	11 (20.0)	11 (24.4)	
T2	37 (67.3)	30 (66.7)	
T3	7 (12.7)	4 (8.9)	
TNM stage			< 0.001[c]
Stage Ⅰ	4 (7.3)	41 (91.1)	
Stage Ⅱ	40 (72.7)	4 (8.9)	
Stage Ⅲ	11 (20.0)	0 (0.0)	
Nerve infiltration			0.591
Absent	12 (21.8)	7 (15.6)	
Present	43 (78.2)	38 (84.4)	
Lymphovascular invasion			0.066
Absent	38 (69.1)	39 (86.7)	
Present	17 (30.9)	6 (13.3)	
Resection region			> 0.999
R0	52 (94.5)	42 (93.3)	
R1	3 (5.5)	3 (6.7)	

CT - determined LN status			0.033[c]
Absent	38 (69.1)	40 (88.9)	
Present	17 (30.9)	5 (11.1)	
Rad-score[b]	0.67 (0.55, 0.77)	0.44 (0.31, 0.62)	< 0.001[c]

Note. Except where indicated, data are numbers of patients, with percentages in parentheses. CA19-9, carbohydrate antigen 19-9 (U/ml); R0, negative surgical margin; R1, positive surgical margin; LNM: lymph node metastasis.

[a] mean ± standard deviation, [b] median (interquartile range), [c] $P < 0.05$ is regarded statistically significant.

Supplemental Table 4. The demographic and pathological characteristics of the clinical utilization cohort.

Variables	dissected LNs ≥ 15 (n = 100)	dissected LNs < 15 (n = 63)	*P* Value
Age (years)[a]	64.0 ± 9.0	63.9 ± 9.8	0.962
Gender			> 0.999
Female	45 (45.0)	28 (44.4)	
Male	55 (55.0)	35 (55.6)	
Tumor location			0.567
Head and neck	79 (79.0)	47 (74.6)	
Body and tail	21 (21.0)	16 (25.4)	
Tumor size (cm)[b]	3.1 (2.2, 3.5)	3.0 (2.5, 3.8)	0.744
CA 19-9 (U/ml)			0.782
≤ 210	49 (49.0)	33 (52.4)	
> 210	51 (51.0)	30 (47.6)	
Total bilirubin (μmol/l)[b]	24.2 (12.3, 123.2)	22.6 (10.1, 155.4)	0.977
Direct bilirubin (μmol/l)[b]	9.6 (4.1, 105.8)	8.1 (3.7, 127.2)	0.980
Albumin (g/l)[b]	44.2 (40.4, 46.0)	43.1 (40.1, 46.4)	0.541
Differentiation			0.010[c]
Well or moderate	82 (82.0)	40 (63.5)	
Poor	18 (18.0)	23 (36.5)	

T stage			0.267
T1	22 (22.0)	8 (12.7)	
T2	67 (67.0)	45 (71.4)	
T3	11 (11.0)	10 (15.9)	
TNM stage			0.370
Stage Ⅰ	45 (45.0)	29 (46.0)	
Stage Ⅱ	44 (44.0)	31 (49.2)	
Stage Ⅲ	11 (11.0)	3 (4.8)	
Nerve infiltration			0.553
Absent	19 (19.0)	15 (23.8)	
Present	81 (81.0)	48 (76.2)	
Lymphovascular invasion			0.151
Absent	77 (77.0)	55 (87.3)	
Present	23 (23.0)	8 (12.7)	
Resection region			0.250
R0	94 (94.0)	62 (98.4)	
R1	6 (6.0)	1 (1.6)	
LNM			0.872
Absent	45 (45.0)	27 (42.9)	
Present	55 (55.0)	36 (57.1)	

Risk-stratification			< 0.001[c]
High-risk	68 (68.0)	22 (34.9)	
Low-risk	32 (32.0)	41 (65.1)	

Note. Risk-stratification was determined by Rad-score calculated by radiomics model according to optimal cutoff value. CA 19-9, carbohydrate antigen 19-9 (U/ml); R0, negative surgical margin; R1, positive surgical margin; LNM, lymph nodes metastasis. [a] mean ± standard deviation, [b] median (interquartile range), [c] $P < 0.05$ is regarded statistically significant.

Supplemental Table 5. Features selected to construct the radiomics model.

Radiomics Features	Coefficient
original_shape_Flatness	0.0735
original_shape_Maximum2DDiameterSlice	0.0391
original_shape_Sphericity	1.420
image_original_glcm_ClusterProminence	1.9614
image_original_glcm_ClusterTendency	0.1653
image_original_glcm_SumSquares	-0.2183
image_original_gldm_SmallDependenceLowGrayLevelEmphasis	-0.2450
image_original_glrlm_ShortRunLowGrayLevelEmphasis	0.1616
image_original_glszm_GrayLevelVariance	-1.1917
image_original_glszm_SmallAreaLowGrayLevelEmphasis	0.9810

Supplemental Table 6. The radiomics quality score: RQS.

**Criteria**	**Point**
1. Image protocol quality-well-documented image protocols (for example, contrast, slice thickness, energy etc.) and/or usage of public image protocols allow reproducibility/replicability	1
2. Multiple segmentations - possible actions are: segmentation by different physicians/algorithms/software, perturbing segmentations by (random) noise, segmentation at different breathing cycles. Analyse feature robustness to segmentation variabilities	0
3. Phantom study on all scanners - detect inter-scanner differences and vendor-dependent features. Analyse feature robustness to these sources of variability	0
4. Imaging at multiple time points - collect images of individuals at additional time points. Analyse feature robustness to temporal variabilities (for example, organ movement, organ expansion/ shrinkage)	0
5. Feature reduction or adjustment for multiple testing - decreases the risk of overfitting. Overfitting is inevitable if the number of features exceeds the number of samples. Consider feature robustness when	3

selecting features	
6. Multivariable analysis with non-radiomics features (for example, EGFR mutation) - is expected to provide a more holistic model. Permits correlating/inferencing between radiomics and non-radiomics features	1
7. Detect and discuss biological correlates - demonstration of phenotypic differences (possibly associated with underlying gene–protein expression patterns) deepens understanding of radiomics and biology	0
8. Cut-off analyses - determine risk groups by either the median, a previously published cut-off or report a continuous risk variable. Reduces the risk of reporting overly optimistic results	1
9. Discrimination statistics - report discrimination statistics (for example, C-statistic, ROC curve, AUC) and their statistical significance (for example, p-values, confidence intervals). One can also apply resampling method (for example, bootstrapping, cross-validation)	2
10. Calibration statistics - report calibration statistics (for example, Calibration-in-the-large/slope, calibration plots) and their statistical significance (for example, P-values, confidence intervals). One	1

can also apply resampling method (for example, bootstrapping, cross-validation)	
11. Prospective study registered in a trial database - provides the highest level of evidence supporting the clinical validity and usefulness of the radiomics biomarker	0
12. Validation - the validation is performed without retraining and without adaptation of the cut-off value, provides crucial information with regard to credible clinical performance	4
13. Comparison to 'gold standard' - assess the extent to which the model agrees with/is superior to the current 'gold standard' method (for example, TNM-staging for survival prediction). This comparison shows the added value of radiomics	2
14. Potential clinical utility - report on the current and potential application of the model in a clinical setting (for example, decision curve analysis).	2
15. Cost-effectiveness analysis - report on the cost-effectiveness of the	0

clinical application (for example, QALYs generated)	
16. Open science and data - make code and data publicly available. Open science facilitates knowledge transfer and reproducibility of the study	1

Supplemental Figures.

Supplemental Figure 1. The calibration curves of the radiomics model in the training cohort (a), validation cohort (b), and testing cohort (c).

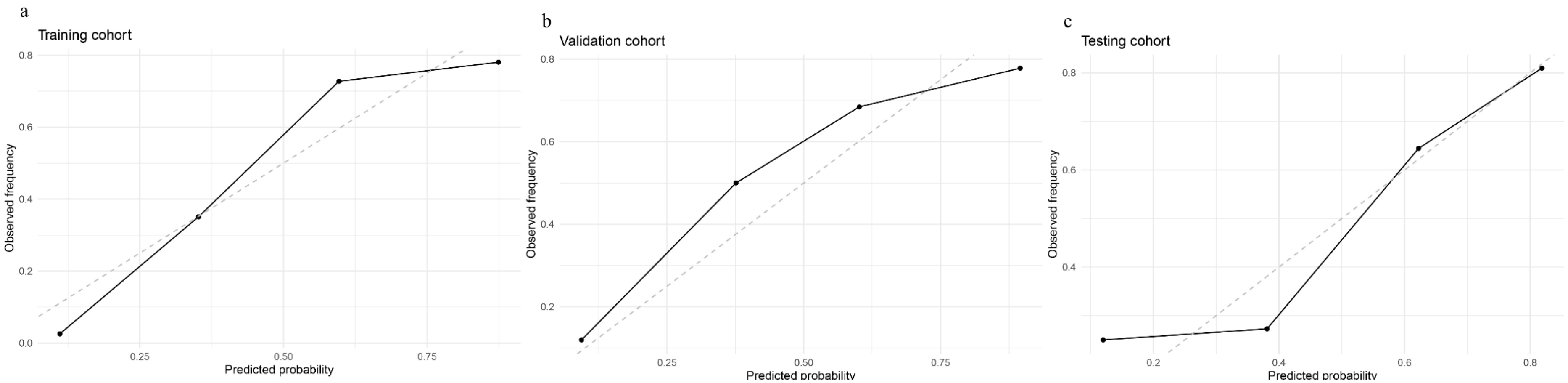

Supplemental Figure 2. The decision curve analysis of the radiomic model in the training cohort (a), validation cohort (b), and testing cohort (c). DCA, decision curve analysis.

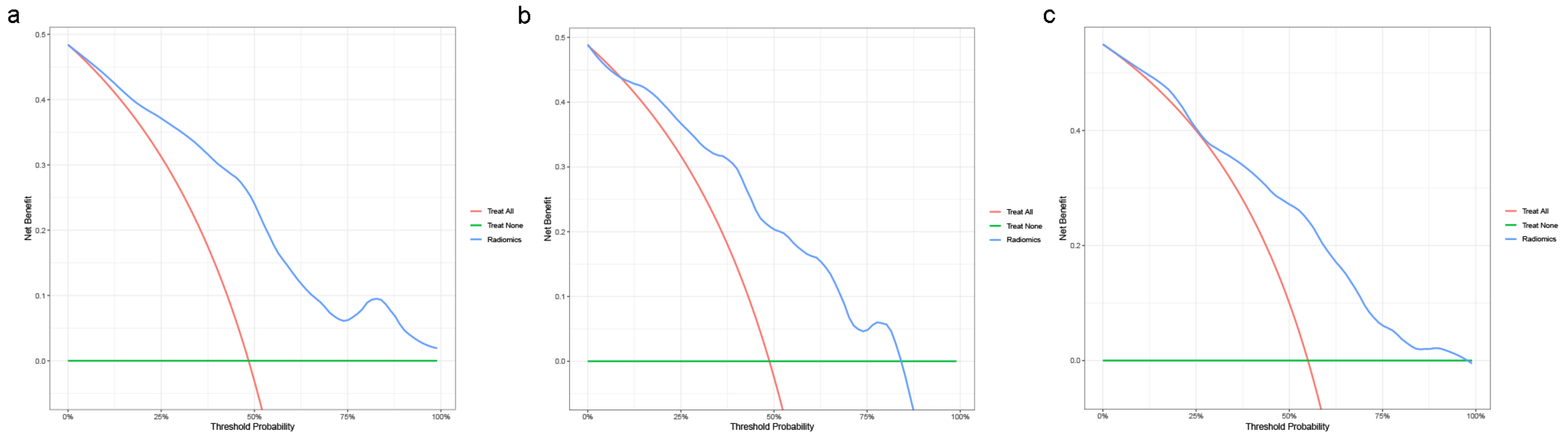

Supplemental Figure 3. Receiver operating characteristic curves of the radiomics model in the tumor size ≤ 3cm subgroup (a), and tumor size > 3cm subgroup (b). AUC, the area under the curve; CI, confidence interval.

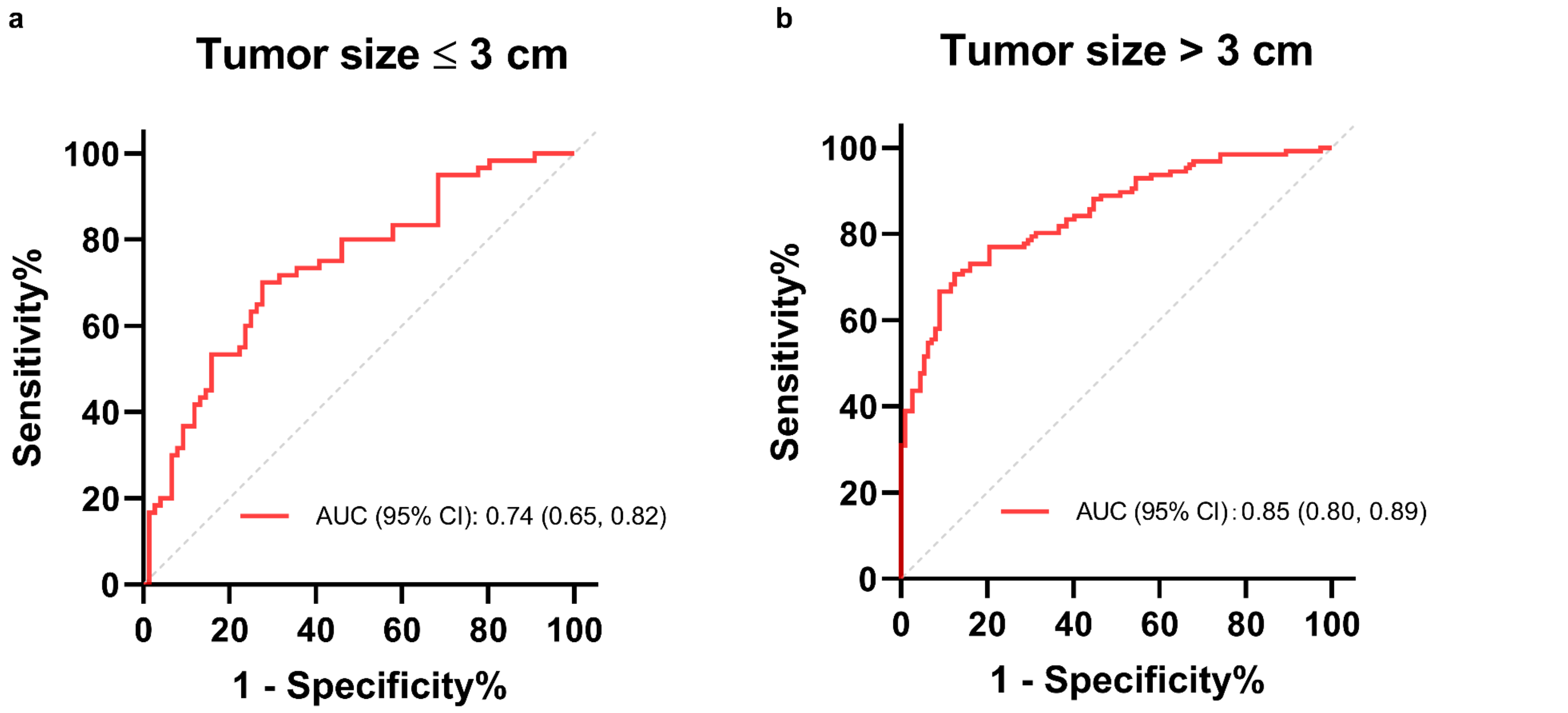

Supplemental Figure 4. Kaplan-Meier analysis of overall survival between different risk of LNM predicted by the radiomics model in the tumor size ≤ 3cm subgroup (a), and tumor size > 3cm subgroup (b). LNM, lymph nodes metastasis. *P* values were calculated using the log-rank test.

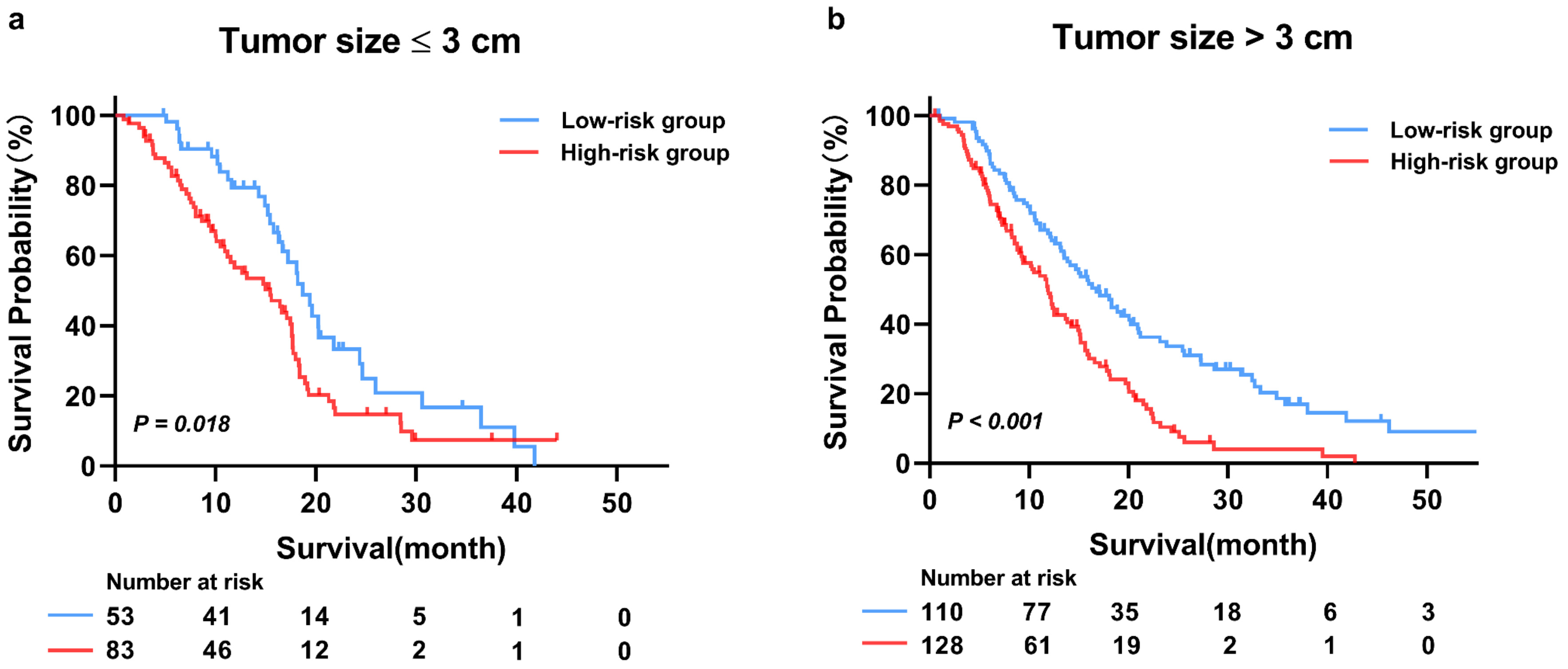

Supplement: Supplementary file 1 — ELECTRONIC SUPPLEMENTARY MATERIAL [file 13244_2025_2025_MOESM1_ESM.pdf]
